# Contributions of the institutions for the nursing professionalization: integrative review (2010-2020) in the light of freidsonian conceptions

**DOI:** 10.1590/0034-7167-2022-0153

**Published:** 2022-11-28

**Authors:** Natália Maria Freitas e Silva Maia, Francisca Aline Amaral da Silva, Agostinho Antônio Cruz Araújo, Ana Maria Ribeiro dos Santos, Fernanda Batista Oliveira Santos, Pacita Geovana Gama de Sousa Aperibense

**Affiliations:** I Universidade Federal do Piauí. Teresina, Piauí, Brazil; II Universidade de São Paulo. Ribeirão Preto, São Paulo, Brazil; III Universidade Federal de Minas Gerais. Belo Horizonte, Minas Gerais, Brazil; IV Universidade Federal do Rio de Janeiro. Macaé, Rio de Janeiro, Brazil

**Keywords:** Nursing, Nursing History, Professional Standardization Organizations, Societies, Sociology, Enfermería, Historia de la enfermería, Organizaciones de Normalización Profesional, Sociedades, Sociología, Enfermagem, História da Enfermagem, Organizações de Normalização Profissional, Sociedades, Sociologia

## Abstract

**Objective:**

To analyze the contributions of the Brazilian Nursing Association and the Federal and Regional Nursing Councils in the literature for the professionalization of nursing in the light of Eliot Freidson’s theoretical conceptions.

**Methods:**

Integrative review of the literature, of socio-professional historical interest, carried out from June to November 2021, through the question: How did professional associations contribute to the professionalization of Brazilian nursing according to the literature from 2010 to 2020? The evidence were organized in a synoptic table, which allowed the construction of a conceptual map.

**Results:**

In the 23 selected studies, the professional associations presented scientific, social and political contributions, which shape the professional field of nursing, enabling knowledge/expertise, autonomy and self-regulation.

**Final considerations:**

The efforts of these institutions reaffirm nursing as an academic and consulting profession, capable of exerting control over the essence of nursing work. They strive to consolidate nursing as a legitimate professional field of health in Brazil.

## RESUMO

**Objetivo:**

Analisar na literatura contribuições da Associação Brasileira de Enfermagem e dos Conselhos Federal e Regionais de Enfermagem para a profissionalização da enfermagem à luz das concepções teóricas de Eliot Freidson.

**Métodos:**

Revisão integrativa, de interesse histórico socioprofissional, realizada de junho a novembro de 2021, mediante a questão: Como as entidades de classe contribuíram para a profissionalização da enfermagem brasileira de acordo com a literatura de 2010 a 2020? Organizaram-se as evidências em quadro sinótico, que possibilitou a construção de mapa conceitual.

**Resultados:**

Nos 23 estudos selecionados, as entidades de classe apresentaram contribuições científicas, sociais e políticas, que conformam o campo profissional da enfermagem, possibilitando conhecimento/*expertise*, autonomia e autorregulação.

**Considerações finais:**

Os esforços dessas entidades reafirmam a enfermagem como profissão acadêmica e de consulta, capaz de exercer controle sobre a essência do trabalho da enfermagem. Elas se empenham em consolidar a enfermagem como legítimo campo profissional da saúde no Brasil.

## RESUMEN

**Objetivo:**

Analizar las contribuciones de la Asociación Brasileña de Enfermería y de los Consejos Federales y Regionales de Enfermería a la profesionalización de la enfermería bajo la luz de las concepciones teóricas de Eliot Freidson

**Métodos:**

Se trata de una revisión integradora, de interés histórico socio profesional, realizada de junio a noviembre de 2021, mediante la pregunta: ¿Cómo han contribuido las entidades de clase con la profesionalización de la enfermería brasileña según la literatura de 2010 a 2020? Las pruebas se organizaron en un cuadro sinóptico que permitió construir un mapa conceptual.

**Resultados:**

En los 23 estudios seleccionados, las entidades de clase presentaron contribuciones científicas, sociales y políticas, que forman el campo profesional de la enfermería y facilitan el conocimiento, la experticia, la autonomía y la autorregulación.

**Consideraciones finales:**

Las entidades reafirman que la enfermería es una profesión académica y de consulta, capaz de ejercer el control sobre la esencia del trabajo en sí y perseveran para consolidarla como un campo profesional legítimo en el Brasil.

## INTRODUCTION

In the process of professionalization of Brazilian nursing, Working class institutions cooperated for the constitution and consolidation of the profession, no longer as an occupation, since recognition as a professional category involves struggles and achievements of leaders in different economic, social and political contexts, contributing to the formation of professional identity^(1)^.

In the scope of the sociology of professions, the American theorist Eliot Freidson states that a profession is distinguished from an occupation by the control it exerts over work^(2)^. The central idea of the sociologist’s proposal is that the most productive method for analyzing professions is based on focusing on the way in which they achieve or lose control over the terms, conditions and content of the work^(2)^.

Furthermore, Freidson characterizes a profession by three aspects: knowledge/expertise, self-regulation and autonomy. Professions share a specialized body of knowledge and qualifications which have jurisdiction, whose credentialing occurs through institutionalization in schools and universities, and occupational control of practice in the labor market, by requiring credentials to perform licensed and state-backed actions^(3)^.

Credentialism is consolidated in the specific knowledge/expertise of the profession, indispensable to legitimize the achievement of autonomy^(1)^. It is through autonomy that the profession controls what is its own^(2)^. When considering these aspects, it is noted that class institutions become bases for profession, discipline and work. They also support professionalism, a set of characteristics specific to professionals^(2)^; and professionalization, characterized by interdependent elements, specialized work based on specific knowledge and ethics, when applying and performing professionally^(1)^.

Class institutions act effectively in favor of the category and society when they defend a political project of training and qualification that meets the interests of professionals in this class and social demands. They promote the dissemination of knowledge about and for the profession, representing these professionals in the different fields of activity in which they are inserted^(4)^.

In Brazil, the vanguard of the organization of nursing and the representation of the categories took place in 1926, with the graduation of the pioneer group of the Anna Nery School of Nursing. Created on August 12 of that year, the National Association of Graduate Nurses (NAGN), currently the Brazilian Nursing Association (ABEn), promoted the improvement of professionals and training programs, with a commitment to growing and consolidating scientific development, social and political aspects of nursing, and its history is permeated by the articulation with other organizations, based on ethical principles and on the attributions that were incumbent upon them^(5-7)^.

ABEn started other nursing organizations and boosted their development in Brazil^(8)^. Thus, the creation of the Federal (Cofen) and Regional Nursing Councils (Coren) has an intrinsic relationship with the history of ABEn. Many efforts were made to implement the creation of the body that would supervise the practice of nursing professionals. The first efforts took place in the period from 1943 to 1947, but only on July 12, 1973, law 5905 was enacted, which provided for the creation of the Cofen/Coren System, an autarchy linked to the Ministry of Labor and Social Security, which has the Disciplinary function of the professional practice of nursing^(8-9)^.

After Cofen and Coren were installed, the discussions on the new Professional Practice Law project, which would replace Law 2604/1955, stood out. Thus, law 7498/1986 was approved, regulated by decree 94406/1987^(10-11)^. Among other Cofen actions, we mention the approval, through Cofen resolution 564/2017, of the new Code of Ethics for Nursing Professionals. In this process, the Cofen Code of Nursing Deontology, from 1976, and the Code of Ethics of Nursing Professionals, from 1993, reformulated in 2000 and 2007^(12)^ were considered.

In this context, the design of the contributions of class institutions to the professionalization of Brazilian nursing makes it possible to understand their influence and relevance to the conformation of nursing as a profession and field of scientific knowledge in health. It also reinforces, through historical facts, the importance of associative life for the construction and strengthening of professional identity.

## OBJECTIVE

To analyze the contributions of the Brazilian Nursing Association and the Federal and Regional Nursing Councils in the literature for the professionalization of nursing in the light of Eliot Freidson’s theoretical conceptions

## METHODS

Integrative literature review, of socio-professional historical interest for nursing, carried out through the following steps: formulation of the problem and the research question; literature search and application of eligibility criteria for the study; selection of articles; reading and extracting relevant data; analysis and interpretation of data; organization of data into categories and presentation of the synthesis of the knowledge produced^(13)^. The bibliographic survey, data collection and analysis took place from June 4 to November 29, 2021. The study adopted the recommendations of The PRISMA 2020 Statement: an Updated Guideline for Reporting Systematic Reviews (PRISMA)^(14)^.

To select the studies, the research question was formulated based on the PICo^(15)^: strategy: class institutions constituted the problem (P); contributions to professionalization, the phenomenon of interest (I); and nursing, the context (Co). Thus, the guiding question was elaborated: How did class institutions contribute to the professionalization of Brazilian nursing according to the literature from 2010 to 2020? This strategy enabled the identification of controlled and uncontrolled descriptors, which were selected by consulting the terms in the Descriptors in Health Sciences (DeCS), Medical Subject Headings (MeSH) and List of Headings of the Cinahl Information Systems.

The bibliographic search was carried out by consulting the electronic databases Cumulative Index to Nursing and Allied Health Literature (Cinahl), Digital Nursing Library (BDENF), Latin American and Caribbean Literature in Health Sciences (Lilacs), Medical Literature Analysis and Retrieval System online (MEDLINE® via PubMed®) and Web of Science. The Boolean operators “OR” and “AND” were used to combine the search terms and intensify the reach of the number of articles that answered the research question, which generated the search strategies (Chart 1) for each of the consulted bases.

**Chart 1:** Databases, number of records and search strategies

Databases, number of records and search strategies
Cinahl (n=498) ( (MH “*Organizations*”) *OR* “*Organizations*” *OR* (MH “*Professional Organizations*”) *OR* “*Professional Organizations*” *OR* (MH “*Nursing Organizations*”) *OR* “*Nursing Organizations*” *OR* “*Brazilian Nursing Association*” *OR* “ABEn” *OR* “*Federal Nursing Council*” *OR* “COFEn” *OR* “*Regional Nursing Council*” OR “COREn” ) AND ( (MH “History”) OR “History” OR “*Aspects, Historical*” *OR* “*Historical Aspects*” *OR* (MH “*History of Nursing*”) *OR* “*History of Nursing*” *OR* “*Nursing, History*” *OR* “*Nursing History*” *OR* “*History Nursing*” ) *AND* “*nursing*”
BDENF (n=219) / Lilacs (n=259) ((mh:(*societies*)) *OR* (*societies*) *OR* (“Associações Profissionais” ) *OR* (“Associações de Profissionais”) *OR* (“Organizações Profissionais”) *OR* (“Organizações de Profissionais” ) *OR* (“Profissionais Associados”) *OR* (*organization, professional*) *OR* (*organizations, professional* ) *OR* (“*Professional Organization*”) *OR* (“*Professional Organizations*”) *OR* (“Asociacion*es Profesionales*”) *OR* (“*Asociaciones de Profesionales*” ) *OR* (“*Organizaciones Profesionales*”) *OR* (“*Organizaciones de Profesionales*” ) *OR* (“*Profesionales Asociados*” ) *OR* (“Associação Brasileira de Enfermagem”) *OR* (ABEn) *OR* (“Conselho Federal de Enfermagem” ) *OR* (cofen) *OR* (“Conselho Regional de Enfermagem”) *OR* (coren ) *OR* (“*Brazilian Nursing Association*”) *OR* (“*Federal Nursing Council*”) *OR* (“*Regional Nursing Council*” ) *OR* (“*Asociación Brasileña de Enfermería*” ) *OR* (“*Consejo Federal de Enfemería*”) *OR* (“*Consejo Regional de Enfermería*”) *OR* (*societies, nursing*) *OR* (“*Nursing Societies*”) *OR* (“*Nursing Society*”) *OR* (s*ociety, nursing*)) *AND* ((mh:(*history*)) *OR* (*history*) *OR* (*aspect, historical*) *OR* (*aspects, historical*) *OR* (“*Historical Aspect*”) *OR* (“*Historical Aspects*”) *OR* (*histories*) *OR* (mh:(“*History of Nursing*”)) *OR* (“*History of Nursing*”) *OR* (“*History Nursing*”) *OR* (“*Nursing History*”) *OR* (*nursing, history*)) *AND* ((mh:(enfermagem)) *OR* (enfermagem) *OR* (*nursings*))
MEDLINE®/PubMed® (n=1.050) (“*societies*”[MeSH Terms] *OR* “*societies*”[*All Fields*] *OR* “*society*”[*All Fields*] *OR* “*society* s”[*All Fields*] *OR* “*societys*”[*All Fields*] *OR* (“*societies*”[MeSH Terms] *OR* “*societies*”[*All Fields*] *OR* (“*professional*”[*All Fields*] *AND* “*organizations*”[*All Fields*]) *OR* “*professional organizations*”[*All Fields*]) *OR* (“*societies*”[MeSH Terms] *OR* “*societies*”[*All Fields*] *OR* (“*organizations*”[*All Fields*] *AND* “*professional*”[*All Fields*]) *OR* “*organizations professional*”[*All Fields*]) *OR* (“*societies*”[MeSH Terms] *OR* “*societies*”[*All Fields*] *OR* (“*organization*”[*All Fields*] *AND* “*professional*”[*All Fields*]) *OR* “*organization professional*”[*All Fields*]) *OR* (“*professional organisation*”[*All Fields*] *OR* “*societies*”[MeSH Terms] *OR* “*societies*”[*All Fields*] *OR* (“*professional*”[*All Fields*] *AND* “*organization*”[*All Fields*]) *OR* “*professional organization*”[*All Fields*]) *OR* “*Brazilian Nursing Association*”[*All Fields*] *OR* “ABEn”[*All Fields*] *OR* “*Federal Nursing Council*”[*All Fields*] *OR* “COFEn”[*All Fields*] *OR* “*Regional Nursing Council*”[*All Fields*] *OR* (“coren”[*All Fields*] *OR* “coren s”[*All Fields*])) *AND* (“*history*”[MeSH *Terms*] *OR* “*history*”[*All Fields*] *OR* “*histories*”[*All Fields*] *OR* “*history*”[MeSH *Subheading*] *OR* (“*history*”[MeSH *Terms*] *OR* “*history*”[*All Fields*] *OR* (“*aspects*”[*All Fields*] *AND* “*historical*”[*All Fields*]) *OR* “*aspects historical*”[*All Fields*]) *OR* (“*history*”[MeSH *Subheading*] *OR* “*history*”[*All Fields*] *OR* (“*historical*”[*All Fields*] *AND* “*aspect*s”[*All Fields*]) *OR* “*historical aspects*”[*All Fields*] *OR* “*history*”[MeSH *Terms*] *OR* (“*historical*”[*All Fields*] *AND* “*aspects*”[*All Fields*]) *OR* “*historical aspects*”[*All Fields*]) *OR* (“*history of nursing*”[MeSH *Terms*] *OR* (“*history*”[*All Fields*] *AND* “*nursing*”[*All Fields*]) *OR* “*history of nursing*”[*All Fields*]) *OR* (“history of nursing”[MeSH *Terms*] *OR* (“*history*”[*All Fields*] *AND* “*nursing*”[*All Fields*]) *OR* “*history of nursing*”[*All Fields*] *OR* (“*nursing*”[*All Fields*] *AND* “*history*”[*All Fields*]) *OR* “*nursing history*”[*All Fields*]) *OR* (“*history of nursing*”[MeSH *Terms*] *OR* (“*history*”[*All Fields*] *AND* “*nursing*”[*All Fields*]) *OR* “*history of nursing*”[*All Fields*] *OR* (“*nursing*”[*All Fields*] *AND* “*history*”[*All Fields*]) *OR* “*nursing history*”[*All Fields*]) *OR* (“*history of nursing*”[MeSH *Terms*] *OR* (“*history*”[*All Fields*] *AND* “*nursing*”[*All Fields*]) *OR* “*history of nursing*”[*All Fields*] *OR* (“*history*”[*All Fields*] *AND* “*nursing*”[*All Fields*]) *OR* “*history nursing*”[*All Fields*])) *AND* (“*nursing*”[MeSH *Terms*] *OR* “*nursing*”[*All Fields*] *OR* “*nursings*”[*All Fields*] *OR* “*nursing*”[MeSH *Subheading*] *OR* “*breast feeding*”[MeSH *Terms*] *OR* (“*breast*”[*All Fields*] *AND* “*feeding*”[*All Fields*]) *OR* “*breast feeding*”[*All Fields*] *OR* “*nursings*”[*All Fields*])
*Web of Science* (n=493) (TS=(*nursing*)) *AND* (TS=(*History*) *OR* TS=(*Aspects, Historical*) *OR* TS=(“*Historical Aspects*”) *OR* TS=(“*History of Nursing*”) *OR* TS=(*Nursing, History*) *OR* TS=(“*Nursing History*”) *OR* TS=(“*History Nursing*”)) *AND* (TS=(*Societies*) *OR* TS=(“*Professional Organizations*”) *OR* TS=(*Organizations*, P*rofessional*) *OR* TS=(*Organization, Professional*) *OR* TS=(“*Professional Organization*”) *OR* TS=(“*Brazilian Nursing Association*”) *OR* TS=(ABEn) *OR* TS=(“Federal *Nursing Council*”) *OR* TS=(COFEn) *OR* TS=(“*Regional Nursing Council*”) *OR* TS=(COREn) *OR* TS=(*Societies,nursing*) *OR* TS=(“*Nursing Society*”) *OR* TS=(*Society, Nursing*) *OR* TS=(“*Nursing Societies*”))

Original studies were included, published in English, Portuguese or Spanish, from January 2010 to December 2020. 2010 was established as the initial year due to the creation of the Scientific Department of Nursing History (DHE) by National ABEn. The DHE is linked to the Center for Studies and Research in Nursing (CEPEn), which has the function of encouraging the development and dissemination of historical research in nursing. The year 2020 was adopted as the final cut, as it has historically impacted society and the profession, professionalism and professionalization of nursing, considering the 2019 coronavirus disease (Covid-19) pandemic. In addition, the World Health Organization (WHO) established this year as the International Year of Nursing and Midwifery^(16)^. Dissertations and theses, case studies, biographies and experience reports were excluded. Also, the articles captured in duplicate were considered only once, keeping them in the specific bases for nursing, followed by the multidisciplinary ones.

It should be noted that two independent reviewers performed the identification, screening, eligibility and inclusion steps. The EndNote reference manager was used to set up the database, identify and eliminate duplicates. Then, the resulting database was exported to the Rayyan Qatar Computing Research Institute (Rayyan QCRI)^(17)^, for screening, eligibility and inclusion of studies. The Rayyan QCRI allows the evaluation of studies with the blinding of the auxiliary reviewer, which favors reliability in the selection of information and methodological precision^(17)^. In the eligibility stage, there was disagreement in 16 studies, which were sent to a third reviewer to issue an opinion on the inclusion or exclusion of the article.

The form adapted from the Occupational Health Nursing Network (RedENSO)^(18)^ instrument was used to extract and record the pertinent information. The evidence of the selected articles was presented in a synoptic table, which enabled the construction of a conceptual map, in order to favor the understanding and discussion of the contributions of class institutions in the professional conformation of nursing. The conceptual map was developed by the American Joseph D. Novak, in the 1970s, and is characterized by the hierarchical or schematic structuring of concepts immersed in a network of propositions, which favors the construction of knowledge. It can be considered as a visual representation to share meanings^(19)^.

For that, we used the free concept mapping software Cmap Tools 6.03 for Windows, due to the ease of handling and construction of interactive diagrams, whose completion occurred with the use of PowerPoint. The conceptual map construct was based on the concepts of knowledge, autonomy and self-regulation, aspects of a profession by Eliot Freidson, placed at the center of the map and highlighted in bold. The dashed box highlights the ABEn entity with contributions to the development of expertise.

Given the methodology used, which was restricted to the use of published data, there was no need for submission to the Research Ethics Committee. The ethical and legal aspects, with regard to authorship, were preserved, and the study did not involve participating subjects at any time.

## RESULTS

**Figure 1:**
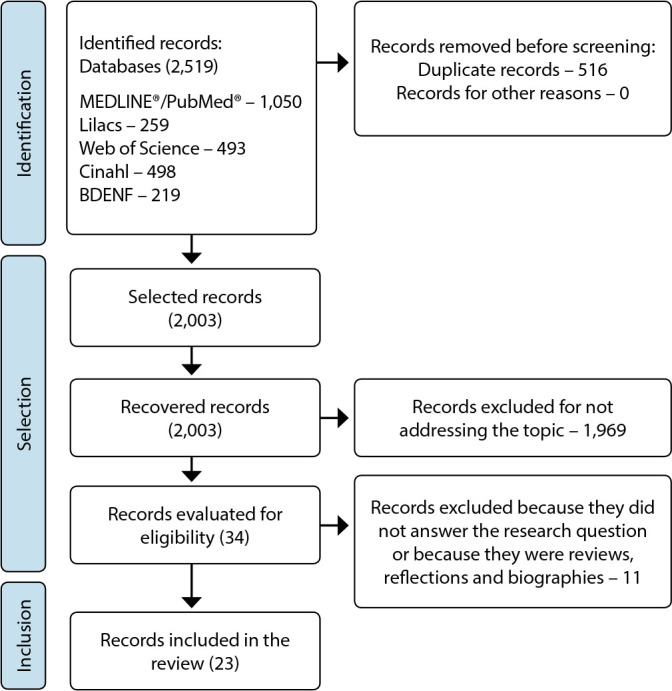
Flowchart for the selection of primary studies included in the integrative review

*Source: Page et al.^(14)^.*

Figure 1 describes the route taken for identification, screening, eligibility and inclusion of studies, according to the databases consulted. Initially, 6318 publications were identified, of which 34 met the eligibility criteria. However, 23 made up the sample.

*Lilacs: Latin American and Caribbean Literature in Health Sciences; Cinahl: Cumulative Index to Nursing and Allied Health Literature; BDENF: Digital Nursing Library.*

The available evidence on class institutions resulted from the final sample of 23 articles^(20-42)^, presented in the synoptic table (Chart 2). Of these, 56% were published in the period from 2015 to 2018^(20-25,34-39,41)^. As for the journals with the highest number of articles on the subject, the History of Nursing Electronic Journal (HNEJ)^(20-21,24-25,32-33,35)^ and the Revista Brasileira de Enfermagem (REBEn)^(23,28-31,41)^ stand out, with about 30% and 26% of production, respectively. All studies used a qualitative approach, of which 78.2% have a historical-social focus.

**Chart 2:** Characterization of studies, according to entity, title, year, journal, design, contributions and Level of Evidence, 2022

Institutions cited	Titles / Years / Periodicals	Designs	Contributions	NE
ABEn	Brazilian nursing in 90 years of associative history: contributions from the Brazilian Nursing Association^(20)^/2018/ Hist enfer Rev eletronica	Exploratory, qualitative	ABEn makes relevant contributions: technical-scientific events, creation of research and scientific publication instances, participation in the regulation and strengthening of the profession, movements in defense of health and exchange with instances of care and teaching management.	VI
ABEn	Higher Education and the Brazilian Nursing Association: contributions to the development and memories of the profession in the West of Santa Catarina^(21)^/2018/ Hist enfer Rev eletronica	Historical and narrative study	The creation of ABEn-SC, Chapeco nucleus, is an important aspect for the development of nursing in western Santa Catarina. The engagement with educational institutions and in knowledge construction movements mark it as an entity of struggles and victories, which seeks professional autonomy.	VI
ABEn	Analysis of the trajectory of the Brazilian Nursing Association - Ceará Section^(22)^/2017/ Rev Rene	Documentary study	ABEn-CE represents the interests of Ceara nursing, when addressing issues related to the teaching of the profession. In addition, it takes a political stand on the struggles of nursing.	VI
ABEn	Associative organization of nursing: struggles for the social recognition of the profession^(23)^ (1943-1946)/2017/ Rev Bras Enferm	Socio-historical, documentary and qualitative study	Sao Paulo’s initiative to create an independent, autonomous unit with its own assets accelerated the process of creating the Association’s sections. The movement to create the section of the Federal District occurred in conjunction with the insertion of the sections in the 1946 statute.	VI
ABEn	Contributions of the Brazilian Nursing Association to Psychiatric Nursing: a look at the Brazilian Nursing Congresses (1947-1981)^(24)^/2016/ Hist enfer Rev eletrônica	Social-historical study	The Brazilian Nursing Congresses contribute to the teaching and care practice of Psychiatric Nursing. Issues between the fields of practice and the qualification of professionals and teaching are discussed.	VI
ABEn	The struggle for the civil organization of nursing in Alagoas: creation of the Brazilian Nursing Association-AL (1962-1965) ^(25)^/2015/ Hist enfer Rev eletrônica	Historical-social, documentary study	Since the beginning, it has been concerned with issues related to teaching, the dissemination of the profession, scientific development, the creation of a staff of nurses in the organizational structure of the State and professional development.	VI
ABEn	Brazilian Nursing Association: 85 years of social responsibility, participation and struggle^(26)^/2014/ Rev enferm UFPE on line	Documentary study	The development of education, the construction and dissemination of knowledge and political participation are two important and interdependent lines of action of ABEn for the social construction of nursing.	VI
ABEn	Pioneering of the Brazilian Nursing Association in the development of research: from the journal to the research center^(27)^/2013/ Esc. Anna Nery	Social-historical study	The creation of its own journal, the organization of National Congresses, the Survey of Nursing Resources and Needs in Brazil, discussions for the development of the profession and the creation of the Center for Studies and Research in Nursing are ABEn’s movements in favor of researches.	VI
ABEn	ABEn and the preservation of professional memory: implementation of the Brazilian Nursing Memory Center^(28)^/2013/ Rev Bras Enferm	Social-historical study	ABEn’s boards are concerned with the preservation of nursing memory, as evidenced by the accumulated, organized and classified documentary mass in the entity’s collection.	VI
ABEn	85 years of ABEn® and 80 years of REBEn® promoting the scientific and professional development of Brazilian Nursing^(29)^/2013/ Rev Bras Enferm	Historical study	ABEn works in the production and dissemination of knowledge and in inter-institutional relations, such as: REBEn, events, national and international relations, leadership in education, participation in the regulation of professional practice and social movements.	VI
ABEn	Brazilian Nursing Association - Sergipe Section: 52 years of will, determination and heart^(30)^/2011/ Rev Bras Enferm	Qualitative study, oral history	History marked by the encouragement of technical-scientific publication, the hiring of nursing professionals by the State, the prohibition of the improper use of the title of nurse, the valorization of the “high standard” nurse, the participation in discussions about the constituent and Health Reform, and struggles for valuing the profession.	VI
ABEn	Brazilian Nursing Association in the context of the 1996 educational reform^(31)^/ 2010/ Rev Bras Enferm	Social-historical study	Strategies of struggle based on the political-expansionist, political-legal and political-organizational guidelines resulting from the 1996 Education Guidelines and Bases Law were drawn up by nursing leaders. In 1994, the National Seminar on Nursing Guidelines was created.	VI
ABEn	Brazilian Association of Graduate Nurses in the context of the Brazil-States alliance: World War II and post-war^(32)^/2010/ Hist enfer Rev eletronica	Historical-social, documentary study	The Association adopts as power strategies the organization of governmental nursing schools, the construction of legislation, the creation of state sections and the holding of Congresses.	VI
ABEn	History of nursing in Campinas: memories of the Brazilian Nursing Association São Paulo Section - Campinas regional^(33)^/2010/ Hist enfer Rev eletronica	Social-historical study	ABEn-Campineira is concerned with the struggles, teaching and creation of nursing schools.	VI
Coren	Contributions of the creation of the technical chamber of obstetrics of the Regional Council of Nursing of Minas Gerais^(34)^/2017/ Enfermagem em Foco	Social-historical study	The creation of the Obstetrics Commission to support the Plenary of Coren-MG aims at the autonomy and recognition of obstetric nursing in changing the care model for normal delivery and in reducing the high rates of cesarean sections in the country.	VI
Coren	Circumstances of installation of the Regional Nursing Council of Alagoas (1973-1978) ^(35)^/ 2016/ Hist enfer Rev eletronica	Social-historical study	In its early years, Coren-AL was dedicated to the organization and maintenance of its operation, with a focus on professional records.	VI
Coren	Nursing profession: its status, that is the question^(36)^/ 2016/ Rev enferm UERJ	Socio-historical study	Credentialism was inherent in the path of the precursors of the profession in the development of Coren-SC. The creation of Coren-SC contributes to the legitimacy and status of the profession.	VI
Coren	The creation of the Regional Nursing Council of Piaui^(37)^/ 2016/ Enfermagem em Foco	Social-historical study	The Special Board carried out the registration of professionals and the election of the first board, which had to clarify professionals about the need for professional registration.	VI
Coren	Regional Nursing Council of Santa Catarina (1975-1986): importance for the profession^(38)^/2015/ Texto Contexto Enferm	Social-historical study	The performance of Coren-SC, from 1975 to 1986, was permeated by difficulties in the adherence of nursing professionals to the purposes of a disciplining and inspection body, which had an impact on the delay of inspection activities.	VI
Coren	Birthplace of the Santa Catarina Regional Nursing Council (1970s) ^(39)^/ 2015/ Rev eletronica enferm	Socio-historical study	The creation of the Council involved ABEn’s commitment and initiative, guaranteeing professional autonomy and power over its work. Assistance and ethical standards in health care are defended.	VI
Coren	Reflections on the trajectory of the Regional Nursing Council of Piaui^(40)^/2013/ Enfermagem em Foco	Documentary, retrospective, descriptive research	It defends the importance of professional regulation and articulates with other institutions to ensure professional valorization and visibility.	VI
ABEn Cofen Coren	Nurses’ militancy in the field of institutionality: versions of the print media^(41)^/2020/ Rev Bras Enferm	Historical study	It was made public about the implementation of Cofen, and the effort with ABEn to update the Law of Professional Practice. It evidenced the creation, election and inauguration of Coren-Bahia, and the supervisory and regulatory character of the profession, in addition to the mandatory registration.	VI
ABEn Cofen Coren	Brazilian Nursing Organization^(42)^/ 2010/ Enfermagem em Foco	Historical study	ABEn mobilizes struggles for growth, development and recognition of the profession, with initiatives in education, research, regulation of professional practice and creation of the Professional Council.	VI

*Note: ABEn: Brazilian Nursing Association; Cofen: Federal Nursing Council; Coren: Regional Nursing Council.*

Each article was evaluated to identify the Level of Evidence (LE), according to the concepts of Melnyk and Fineout-Overholt, classified into six levels: I for systematic review, meta-analysis or studies from guidelines based on randomized controlled clinical trials; II for at least one randomized controlled clinical trial; III for controlled studies without randomization; IV for case-control or cohort studies, as long as they are well designed; V for systematic review of qualitative and descriptive studies; VI for a single qualitative or descriptive study; VII for the opinion of authorities and/or expert committee reports^(43)^. Publications were classified in Level of Evidence VI, resulting from a descriptive or qualitative study.

The ABEn entity was present exclusively in 61% of the studies^(20-33)^, and the Cofen/Coren in 30%^(34-40)^; 9% of the articles dealt with these nursing institutions^(41-42)^.

**Figure 2:**
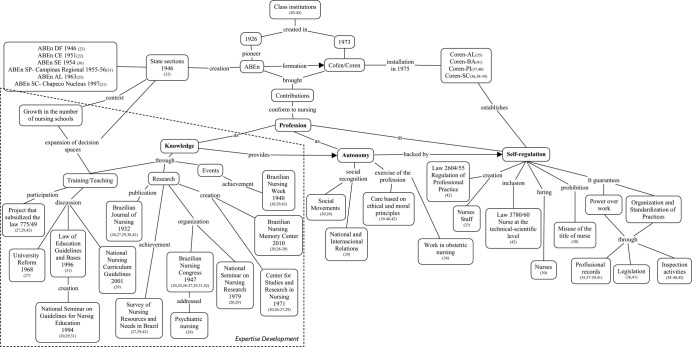
Conceptual map on the contributions of the Brazilian Nursing Association and the Federal and Regional Councils of Nursing in the professional formation of nursing

*Note: ABEn -Brazilian Nursing Association; ABEn DF - Brazilian Nursing Association Distrito Federal Section; ABEn CE - Brazilian Nursing Association Ceara Section; ABEn SE - Brazilian Nursing Association Sergipe Section; ABEn SP - Regional Campinas - Brazilian Nursing Association São Paulo Section Campinas Regional; ABEn AL - Brazilian Nursing Association Alagoas Section; ABEn SC - Nucleo Chapecó - Brazilian Nursing Association Santa Catarina Section, Chapeco Nucleus. Cofen/Coren - Federal Nursing Council/ Regional Nursing Council; Coren-AL -Regional Nursing Council of Alagoas; Coren-BA - Regional Nursing Council of Bahia; Coren-PI- Regional Nursing Council of Piauí; Coren-SC - Regional Nursing Council of Santa Catarina.*

These evidence were schematized in a conceptual map (Figure 2), in order to present how the different contributions of the ABEn and Cofen/Coren class institutions shape the professional field of nursing, according to the concepts of the theoretical Eliot Freidson.

## DISCUSSION

Class institutions aim and mobilize the strengthening of nursing^(1)^, considering the criteria that distinguish a profession from an occupation, namely: own knowledge and skills, autonomy and self-regulation and especially reliable members^(1,3)^. In this context, it is clear that the publications from 2010 to 2020 reinforce the contributions of ABEn and the Cofen/Coren system in professional conformation. The grouping of scientific capital, as proposed by the DHE, promotes and preserves the history of nursing in different contexts, contributing to the dissemination of knowledge that permeates the historical process of the profession and its professionalization.

The construction of nursing as a science and a profession result from the constitution of its own field of knowledge and a scientific framework to support and sustain professional practice. This constitution is permeated by the trajectory of different subjects in different contexts, whose appreciation of history, encouraged by the DHE, contributes to the construction of professional identity. Historically, ABEn is the principle behind the design and production of this knowledge for nursing, with initiatives that involve issues related to nursing education and training, scientific research and its propagation among professionals^(20,24,26-30,31,42)^.

Concerning nursing education and training, in the 1940s, ABEn created the project that subsidized law 775/1949 and institutionalized nursing education in Brazil. At the end of the 1960s, she discussed the implications of the University Reform for the training of nurses^(27,29,42)^. These facts show efforts to establish and dominate ownership over a specific field of knowledge, the acquisition of which needs to be taught and perfected, through the structuring of a curriculum that includes the know-how of the profession.

In the context of expanding nursing schools in Brazil and in the proposal to form another affiliated association (in the 1940s)^(23,32)^, ABEn, in the 1946 Statute, created state and regional sections^(21-23,25,30,33)^. The initiative to form these sections reinforces not only the maintenance of the Brazilian nursing unit, but also the expansion and mastery of decision-making spaces in nursing education and training, being present in the most diverse Brazilian regions. Maintaining the nursing unit was important for its strengthening, since ABEn was the only existing entity with the power to represent Brazilian nursing until the creation of Cofen^(44)^.

Still in the field of education, the 1990s registered two important historical moments for nursing with the involvement of ABEn: the participation of the Education Boards, providing subsidies to the Federal Council of Education, for the approval of the new minimum nursing curriculum, in 1994. In the context of the educational reform that defines the approval of the most important Brazilian law, with regard to education, the Law of Directives and Bases of National Education (LDB), in 1996, in which ABEn undertook strategic efforts in struggles related to to political-expansionist, political-legal and political-organizational guidelines^(31)^. In the midst of this process, ABEn held the National Seminars on Guidelines for Nursing Education (SENADEns)^(20,29,31)^, which discussed and encouraged the elaboration and approval of the National Curriculum Guidelines, approved at the turn of the 21st century, in 2001. From then on, ABEn turned to its own implementation and consolidation^(29)^.

The process of professionalization of an occupation mainly involves the political processes which occupations obtain the State to grant them the exclusive right to perform certain tasks, to recruit and educate members, to delimit the field of action in relation to other occupations and to define in which the work will consist. The State plays a decisive role in the success of a profession, formally granting it the right to monopolize professional practice in the labor market, inhibiting unfair competition and imposing boundaries of competence^(45)^.

ABEn, working together with the Ministry of Education and the Federal Council of Education in the context of the Brazilian educational reform, plays a leading role in directing and reinforcing the formation of expertise necessary for the performance of nursing. Once directed to the production of knowledge and the domain of qualification, there is control of work and ownership of a body of formal knowledge learned in higher education institutions, in other words: the knowledge and qualification necessary for the exercise of the work for the profession.

Other efforts by ABEn to appropriate the knowledge necessary for nursing and the improvement of this knowledge are recognized, such as the movements for the development of research^(27)^. In this regard, the dissemination of knowledge produced through the publication of the Revista Brasileira de Enfermagem (REBEn)^(20,27,29-30,42)^, created in 1932 under the name Annaes de Enfermagem, stands out. It is configured as a space for the formation of the scientific community, as well as for the structuring of the scientific field. Therefore, it reveals the scientific thinking of Brazilian nursing and ABEn’s associative life policy^(29)^. REBEn is the materialization of the desire to solidify nursing science knowledge for the collective.

The development of scientific activities is also noteworthy as shown in the conceptual map. It begins with the production itself of the first research on the numerical and qualitative situation of Brazilian nursing^(27,29,42)^, which permeates the organization of spaces for discussion and scientific dissemination of nursing in the national and international scenario^(20,24,26-29,31,42)^ and immortalizes memories, through the preservation of the material and symbolic heritage of nursing. This is the Brazilian Nursing Memory Center, a place where a dense mass of documents accumulated over time is kept, which reveals the preservation of the memory of those who make the association^(28)^. The exchange of scientific knowledge and nursing practices is also provided by the agenda of the Brazilian Nursing Week, a heritage of ABEn^(20,29,42)^.

Professional knowledge establishes the authority to have control over one’s work, as described by the sociologist Freidson: “authority of imputed expertise”, that is, professionalization requires the establishment of an authority of expertise, a professional knowledge that is so complex and incomprehensible by lay people, which lends relevance to the work, as well as the recognition of the superiority of knowledge and competence, guaranteeing technical autonomy^(45)^. This is the case of the specific themes discussed at the Brazilian Nursing Congresses (CBEn). Among these, psychiatric nursing is mentioned, which addresses aspects of the field of practice, professional qualification, as well as conceptual bases for the teaching of psychiatric Nursing^(24)^.

It is observed that ABEn contributes to the scientific knowledge of nursing, in order to favor the development of competences for the profession. Thus, it makes it possible to ensure that, in addition to increasing expertise, these professionals have autonomy in their professional practice.

Thus, nursing autonomy is legitimized by the implementation of theories, as well as care based on the systematization of nursing care^(46)^. It is understood that nursing is a characteristically autonomous profession, capable of independently exercising its own work in different scenarios. Its object of care is related to human responses and processes of adaptation to situations of health problems, not just compliance with the prescriptions of other professionals who make up the multidisciplinary team.

It is up to nurses to achieve this autonomy, supported by their own knowledge and recognition of the positive value of the profession for society^(3)^. Professional autonomy is not obtained by the profession, but results from a political and social process of legal concession, which is conferred on the profession by society^(45)^. From this perspective, in many of the immense care voids in the Brazilian territory, nursing is present in society. It autonomously performs the care, aiming at the promotion, protection and recovery of the population’s health. The growth of entrepreneurship in nursing and the increase in nursing offices are also emphasized, which reaffirms the autonomy of this profession. Therefore, it cannot be detached from social transformations to reinforce its autonomy and necessity.

In this context, ABEn engages in the most different movements of society. It participates in the fight in defense of the Unified Health System - *Sistema Único de Saúde* (SUS), of quality health care for citizens, of laws and public social and health policies, of the consolidation of nursing as a social practice. It is present in municipal and state councils and health conferences^(20,26)^.

In addition to social movements, ABEn maintains national and international relations to represent Brazilian nursing. Thus, it participates in the International Council of Nurses (ICN), the Asociación Latinoamericana de Escuelas y Facultades de Enfermería (Aladefe) and the Federación Panamericana de Profissionales de Enfermería (Feppen)^(29)^. It is believed that the articulation of ABEn with other institutions in the world of nursing makes the mission for Brazilian nursing even more expressive. The involvement, under the responsibility of the International Council of Nurses, in the creation of the International Classification of Nursing Practices (ICNP®) and the International Classification of Nursing Practices in Collective Health (CIPESC®)^(29)^, both instruments aimed at support the systematization of nursing care and, consequently, the autonomy of the profession.

In order to have control over its own work, in addition to investing in knowledge and technical-scientific improvement, ABEn fights to obtain appreciation and regulation of the profession, which has already resulted in laws 2604 of 1955^(42)^ and 3780 of 1960, which include the nurse at the technical-scientific level^(42)^. In addition, there are efforts to build a Nurse staff^(25)^, hire a nursing professional to replace people without specific training, initiatives to prohibit the misuse of the nursing title^(30)^ and discussion of private actions for nurses^(33)^. These facts show the concern to regulate and regulate the exercise of the profession by those who possess the necessary expertise.

ABEn actively participated in the formation of the legal provisions of the profession, with the creation of a body that self-regulated the practice of nursing and, consequently, protected members and society by guaranteeing the aptitude of those who performed it^(20,29,42)^. Cofen/Corens were created, which granted legitimacy and autonomy to the exercise and specific practice of the profession, in addition to valuing assistance based on ethical and moral principles^(39-40,42)^.

Thus, contributions from Corens, such as Minas Gerais, where there was an effort to develop the Technical Chamber of Obstetrics (CTO), in 2007, aim to guarantee autonomy. The creation and mobilization of the CTO has a social and political impact on the valorization of obstetric nursing by bringing professional recognition of obstetric nurses, not only in the service in which they work, but also for the whole society^(34)^.

Recognition comes from the autonomy necessary to achieve professional status. The reach and maintenance of this status depends on the protection of society, which is convinced of the special value of work^(3)^. Thus, the councils help society to trust and feel secure with the work offered. Thus, they safeguard and protect the members and the population. In this context, the current movements of Cofen/Corens and ABEn are emphasized to ensure dignified conditions for professional practice, having as the agenda for discussions and actions the law of the minimum wage for nursing and the 30-hour working day. In addition, the movements seek to guarantee quality training and committed to the development of professional skills, by rejecting undergraduate nursing courses in the distance modality.

The protection granted to members and the population results from the profession’s ability to carry out self-regulation, understood as control of the exercise of expertise carried out by professional associations that hold legal and formal authority granted by the State^(2)^. The institution of this inspection body was important for the nursing profession, as ABEn has a representative role, but does not have the legal rights to carry out professional registration^(44)^.

Therefore, through the Cofen/Corens system, nursing obtains control of a specific work domain, necessary to achieve autonomy^(2)^. In addition, it guarantees power over work, in addition to organizing, regulating and ensuring the quality of the practice of the profession, through professional registration^(35,37-39,41)^, legislation^(36,41)^ and inspection activities^(39-40,42)^. These actions allow it to be practiced by those who have specialized, qualified knowledge and qualifications granted for the exercise, that is, the credential, defending it regarding the quality of care and ethical standards, which favors visibility and recognition^(36-39)^.

Cofen and Corens work in the development of this professional class at a local and regional level^(34-41)^. Thus, the existence of an institution that regulates the exercise is essential for the credentialism and self-affirmation of the profession.

The achievements historically built by nursing class institutions become even more evident in the year 2020, considering the pandemic context of Covid-19. In the midst of the health crisis, nursing once again reaffirms its protagonism, professional practice through knowledge/expertise, self-regulation and autonomy. Professionalism is highlighted in the health scenario and clearly confirms the social need for nursing.

### Limitations of the study

In this review, the searches were carried out in databases directed to the area of health and nursing. However, the lack of research in databases in the area of history and cross-search may not have covered all articles on the subject.

In addition, the study did not cover the analysis of historical facts implemented by all sections of ABEn, which could also represent a contribution to the conformation of specific or specific knowledge of nursing in a certain region, considering the plurality of the population and Brazilian regions.

### Contributions to the area of nursing and teaching the history of nursing

Class institutions must be strengthened by knowledge and recognition of the contribution to the credentialism of nursing as a profession. From this perspective, many achievements permeate ABEn and Cofen/Coren, and it is important to socialize them. The findings can serve as an incentive to carry out studies that individually address regional nursing sections. Thus, they will highlight, deepen the specificities and expand the knowledge and expertise of each region, so that the actions result in the valorization and professional recognition of autonomy, considering that Brazil is a continental country.

Furthermore, making these contributions visible is a relevant agenda to awaken nursing professionals to the importance of associative life, to increase the status quo and understanding of nursing as a practice and social need. The institutions provide an opportunity to expand the debates about the monitoring of professionals in the transformations of the country’s political and social processes, mainly in the guarantee of universal rights to health.

## FINAL CONSIDERATIONS

The studies included in this review, supported by Fredsonian conceptions, revealed that the nursing institutions Brazilian Nursing Association and Federal and Regional Councils of Nursing contribute to the professionalization process, supported by their own knowledge and the demarcation of expertise, linked to institutionalization, autonomy and self-regulation.

The Brazilian Nursing Association was categorical in the struggles and actions that configure the development of expertise through acting in nursing education/education, in the increment of research and in the dissemination of this knowledge through the various forms of socialization. The conformation of this expertise strengthens the delimitation of the areas of necessary competences and skills, differentiating them from other professions. Through the efforts of the Brazilian Nursing Association, the council was created. Thus, there is the emergence of a body that regulates and supervises the exercise of the profession and its self-regulation, supporting the credentials for professional autonomy.

It is concluded that the continuous efforts undertaken by these class institutions reverberate in the year 2020 when nursing, in the international year by the World Health Organization, stands out in the leading role in facing the pandemic. It shows itself with technical, scientific, ethical, humanistic and professional competence, confirming its function as an academic and consulting profession, whose practice for the community is based on scientific knowledge produced by peers. Thus, nursing is reinforced as a qualified profession, capable of exercising control over its own work, autonomously. Thus, in different historical contexts, these institutions legitimize Nursing as a health profession in Brazil.
